# Pancreas Ductal Adenocarcinoma and its Mimics: Review of Cross-sectional Imaging Findings for Differential Diagnosis

**DOI:** 10.5334/jbsr.1644

**Published:** 2018-10-26

**Authors:** Seung Soo Kim, Gyo Chang Choi, Sung Shick Jou

**Affiliations:** 1 Soonchunhyang University College of Medicine, Cheonan Hospital, KR; 2 Soonchunhyang University Gumi Hospital, KR

**Keywords:** Pancreatic neoplasms, Pancreatitis, Multidetector computed tomography, Magnetic resonance imaging, Differential diagnosis

## Abstract

Ductal adenocarcinoma is the most common pancreatic neoplasm. A variety of pancreatic lesions mimic pancreas ductal adenocarcinoma (PDAC), such as high-grade neuroendocrine tumors, small solid pseudopapillary tumors, metastases, focal autoimmune pancreatitis, and groove pancreatitis. These occasionally look similar in images, but they have differential diagnosis points. Familiarity with the imaging features of PDAC and its mimics is paramount for correct diagnosis and management of patients. In this essay, we describe imaging findings of PDAC and its mimics for differential diagnosis.

## Introduction

Pancreas ductal adenocarcinoma (PDAC) accounts for 85–90% of all pancreatic neoplasms and is one of the leading causes of death worldwide [1,2]. Various other tumors and inflammatory lesions in the pancreas occasionally mimic PDAC. It is clinically important to differentiate PDAC from other pancreatic lesions because of different prognosis and treatment options [3,4,5]. PDAC commonly involves various major vessels around the pancreas and frequently accompanies distant metastases. As a result, less than 20% of patients with PDAC are eligible for resection at the time of diagnosis [6]. On the other hand, patients with neuroendocrine tumors (NETs) or solid pseudopapillary tumors (SPTs) are usually good candidates for surgical resection and show better prognosis than those with PDAC [3,7]. Moreover, most inflammatory lesions do not need surgical resection but rather conservative treatment [8,9]. Therefore, the objective of this article is to assist in differential diagnosis by describing imaging features of PDAC and its mimics.

## Imaging Features of PDAC

**Figure 1:**
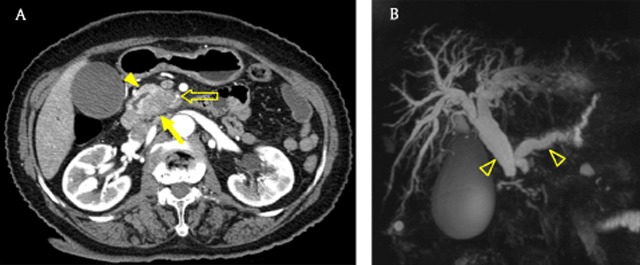
A 79-year-old woman with pancreatic ductal adenocarcinoma. **(A)** Axial pancreatic phase CT image shows lower attenuating mass (arrow) compared with the pancreas parenchyma (arrowhead) in the pancreatic head, which encases the first jejunal branch of the superior mesenteric artery (open arrow). **(B)** Magnetic resonance cholangiopancreatography (MRCP) image shows dilatation of the bile and pancreatic ducts, the so-called double duct sign (open arrowheads). Note the abrupt narrowing of both bile and pancreatic ducts.

**Figure 2:**
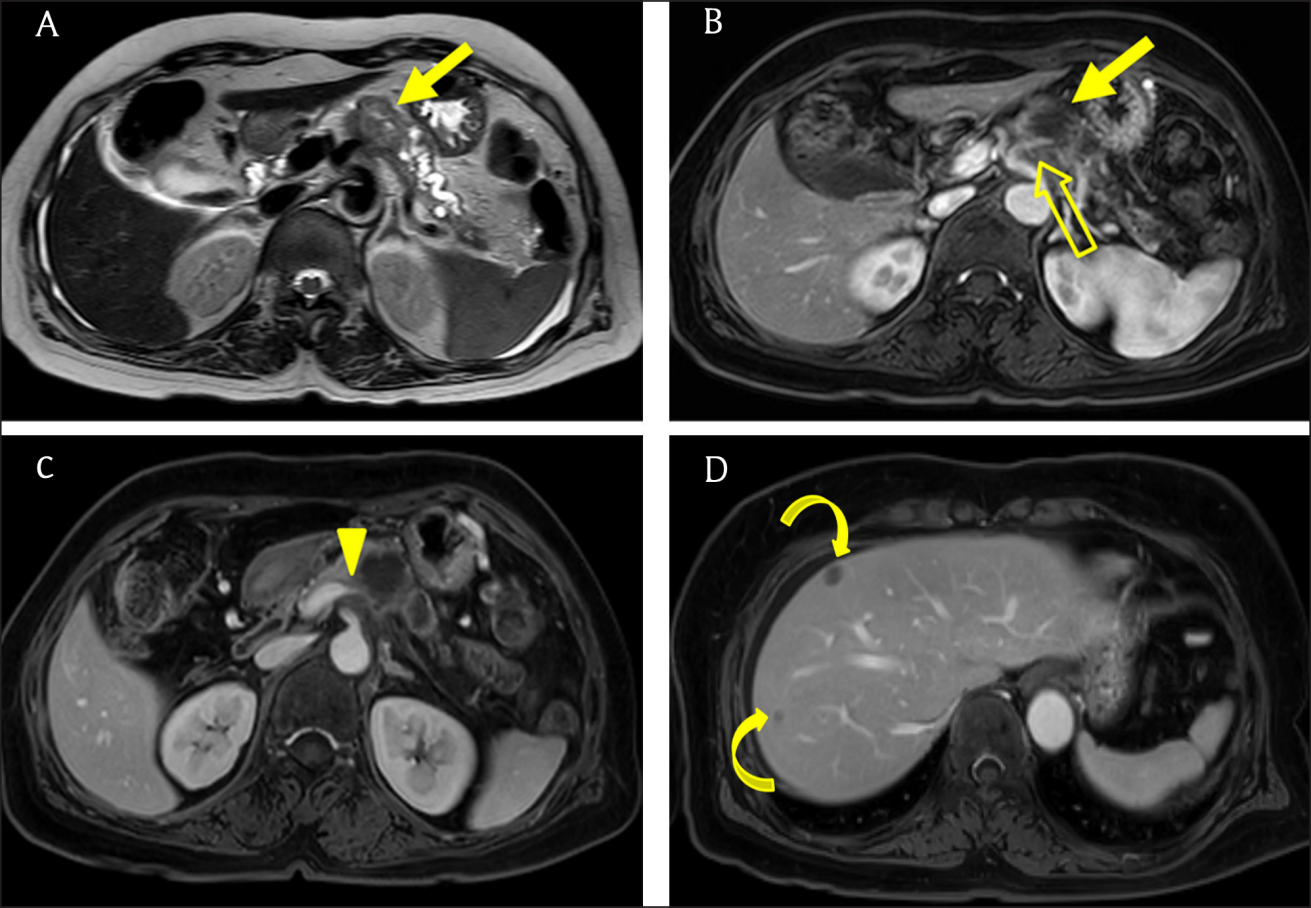
A 60-year-old woman with pancreatic ductal adenocarcinoma. **(A)** Axial T2-weighted image shows a hyperintense mass (arrow) in the pancreatic body with upstream dilatation of the pancreatic duct. **(B, C)** Axial gadoxetic acid-enhanced arterial and portal venous phase MR images show a hypovascular mass (arrow) that invades the splenic artery (open arrow) and vein (arrowhead). **(D)** Axial portal venous phase MR image shows hypovascular metastatic lesions (curved arrows) in the liver.

Computed tomography (CT) is a useful modality for detecting and staging PDACs [10]. PDACs usually appear as low attenuating masses in the pancreatic and portal venous phases and typically accompany pancreatic duct dilatation with abrupt narrowing (Figures 1A, 1B, and 2A). Bile duct dilatation is occasionally combined with a dilated pancreatic duct in cases of pancreas head cancer; this is called the double duct sign (Figure 1B) [11]. Vascular invasion is important for diagnosing PDAC and determining therapeutic options and is supposed when there is a vascular caliber change, irregular vessel wall, more than 180° of vessel is in contact with the tumor, or peritumoral fat infiltration is identified (Figures 1A, 2B, and 2C) [12,13]. Liver metastases from PDAC usually show hypovascularity (Figure 2D).

## Imaging Features of Neoplastic Mimics

### High-grade neuroendocrine tumors

**Figure 3:**
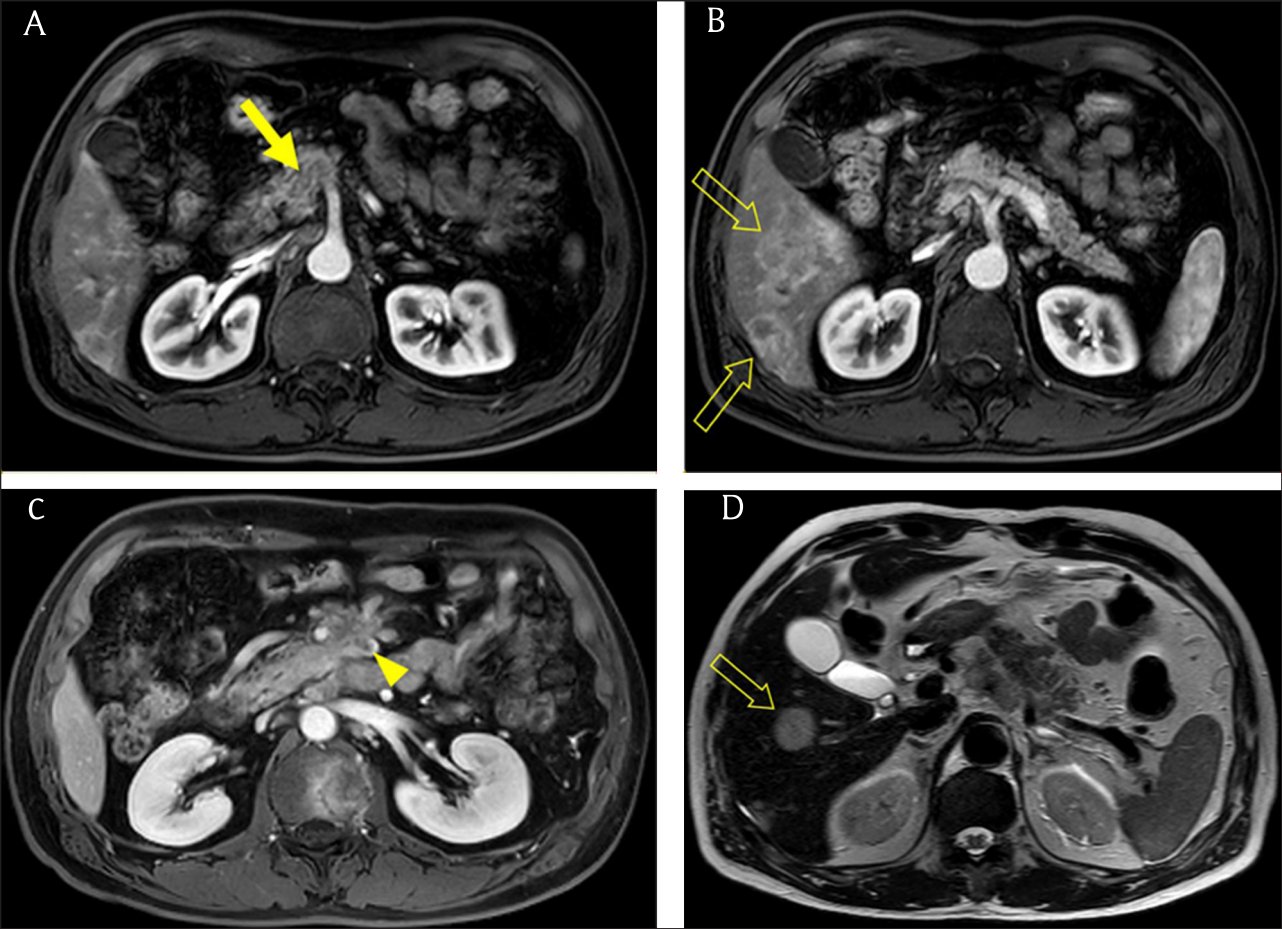
A 60-year-old man with a high-grade neuroendocrine tumor. **(A, B)** Axial gadoxetic acid-enhanced arterial phase MR images show an irregular mass (arrow) in the uncinate process of the pancreas. There are hypervascular masses (open arrows) in the right hepatic lobe. **(C)** Axial portal venous phase MR image shows tumor thrombosis (arrowhead) in a branch of the superior mesenteric vein. **(D)** Axial T2-weighted image shows a hyperintense mass (open arrow) in the right hepatic lobe. There is no dilatation of the pancreatic duct.

Pancreatic NETs originate from the islet cells of Langerhans and are divided into low-, intermediate-, and high-grade according to the World Health Organization classification [5]. High-grade NETs more frequently show vascular invasion, lymph node metastasis, and diffusion restriction compared with low-grade; therefore, high-grade NETs can mimic PDAC on images (Figure 3A) [7,14,15]. However, high-grade NETs usually do not show pancreatic duct dilatation. In addition, they occasionally accompany tumor thrombus, which can be helpful in the differential diagnosis of high-grade NETs from PDAC (Figure 3C) [16]. Liver metastases from NETs frequently reveal findings of hypervascularity and intralesional hemorrhage, in contrast to those from PDAC, which reveal hypovascularity (Figure 3B and 3D) [17,18].

### Small (≤3 cm) solid pseudopapillary tumors

**Figure 4:**
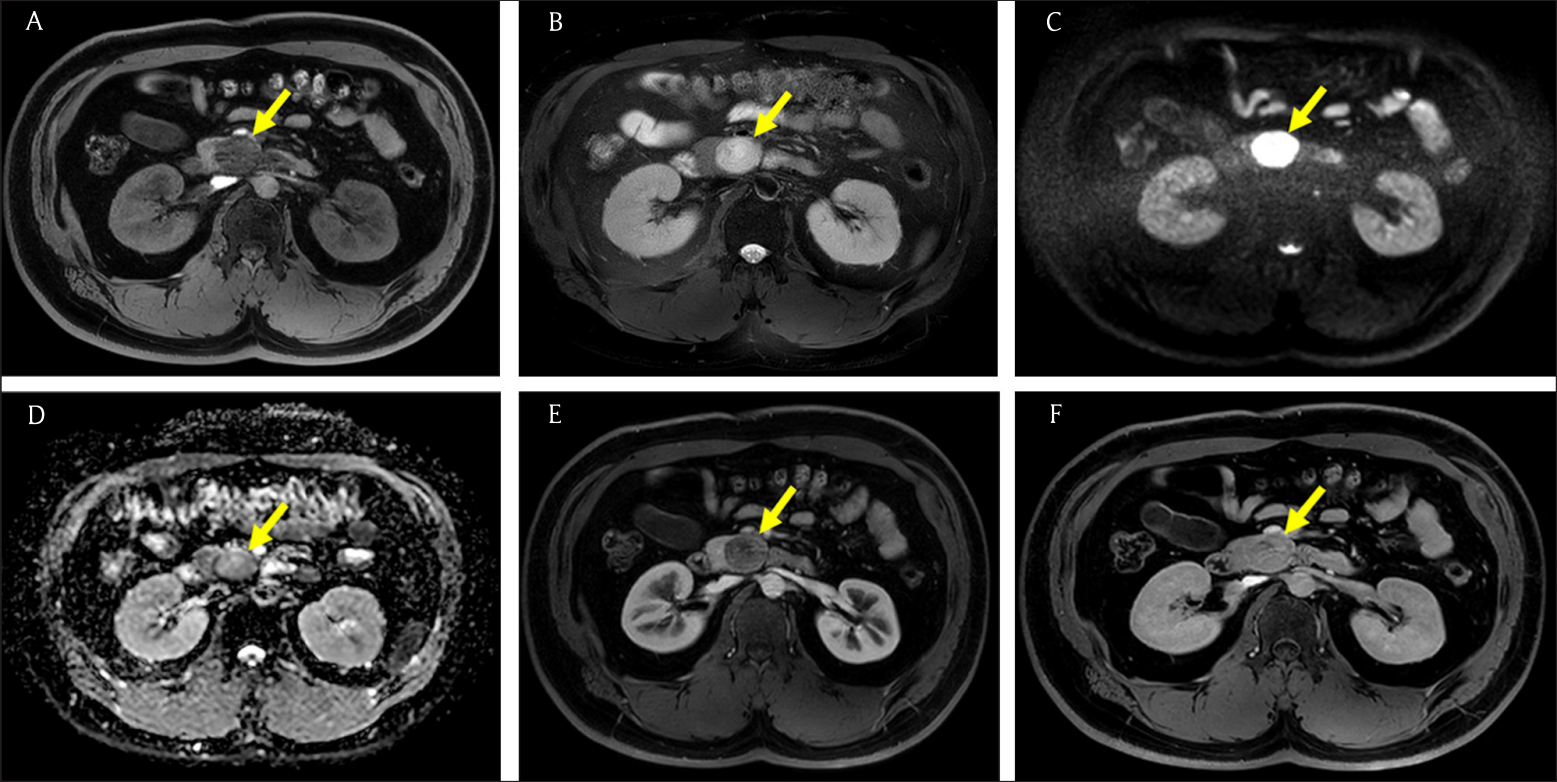
A 31-year-old man with a small solid pseudopapillary tumor. **(A)** Axial T1-weighted fat-suppressed MR image shows a 3-cm solid mass (arrow) with a well-defined margin in the pancreatic head. **(B)** On axial T2-weighted fat-suppressed MR image, the mass (arrow) shows relatively homogeneous high signal intensity. **(C, D)** Axial diffusion-weighted image (b = 800 s/mm^2^) and apparent diffusion coefficient map show the mass (arrow) with diffusion restriction. **(E, F)** Axial gadolinium-enhanced arterial and 3-minute delayed phase MR images show early heterogeneous and progressive enhancement of the mass (arrow).

SPTs are uncommon neoplasms with low malignancy potential, occurring predominantly in young women [3,19]. Calcification, cystic change, and internal hemorrhage due to weak vascular channels are characteristic features of SPT [20,21]. However, small (≤3 cm) SPTs show different imaging findings from larger ones, primarily a homogeneous nature. Small SPTs show a pure solid consistency, well-defined margin, and diffusion restriction on magnetic resonance (MR) imaging (Figure 4A–D) [22,23]. After contrast infusion, small SPTs reveal an early heterogeneous nature, followed by a progressive enhancement pattern (Figure 4E and 4F) [22,23].

### Metastases

**Figure 5:**
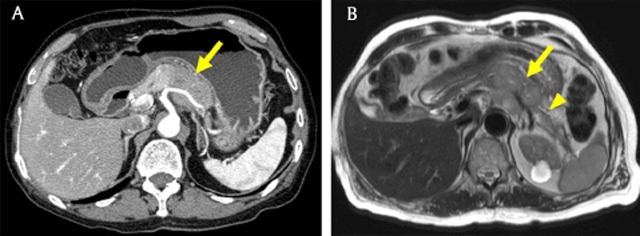
A 67-year-old man with prostate cancer. **(A)** Axial pancreatic phase CT image shows a low attenuating mass (arrow) encasing the splenic artery in the pancreatic body. **(B)** Axial T2-weighted image shows hyperintense mass (arrow) and normal pancreatic duct (arrowhead) without upstream dilatation.

A previous study [24] reported that up to 11% of patients with malignancy have pancreatic metastases at autopsy. In patients with another malignancy who have pancreatic mass, lack of pancreatic duct dilatation usually suggests that pancreatic metastases are more likely than PDAC (Figure 5) [25]. However, pancreatic ductal involvement can unfortunately occur in some pancreatic metastases. In those cases, the absence of adjacent vascular invasion may be a clue for differentiation of metastases from PDAC [24,26].

## Imaging Features of Inflammatory Mimics

### Focal autoimmune pancreatitis

**Figure 6:**
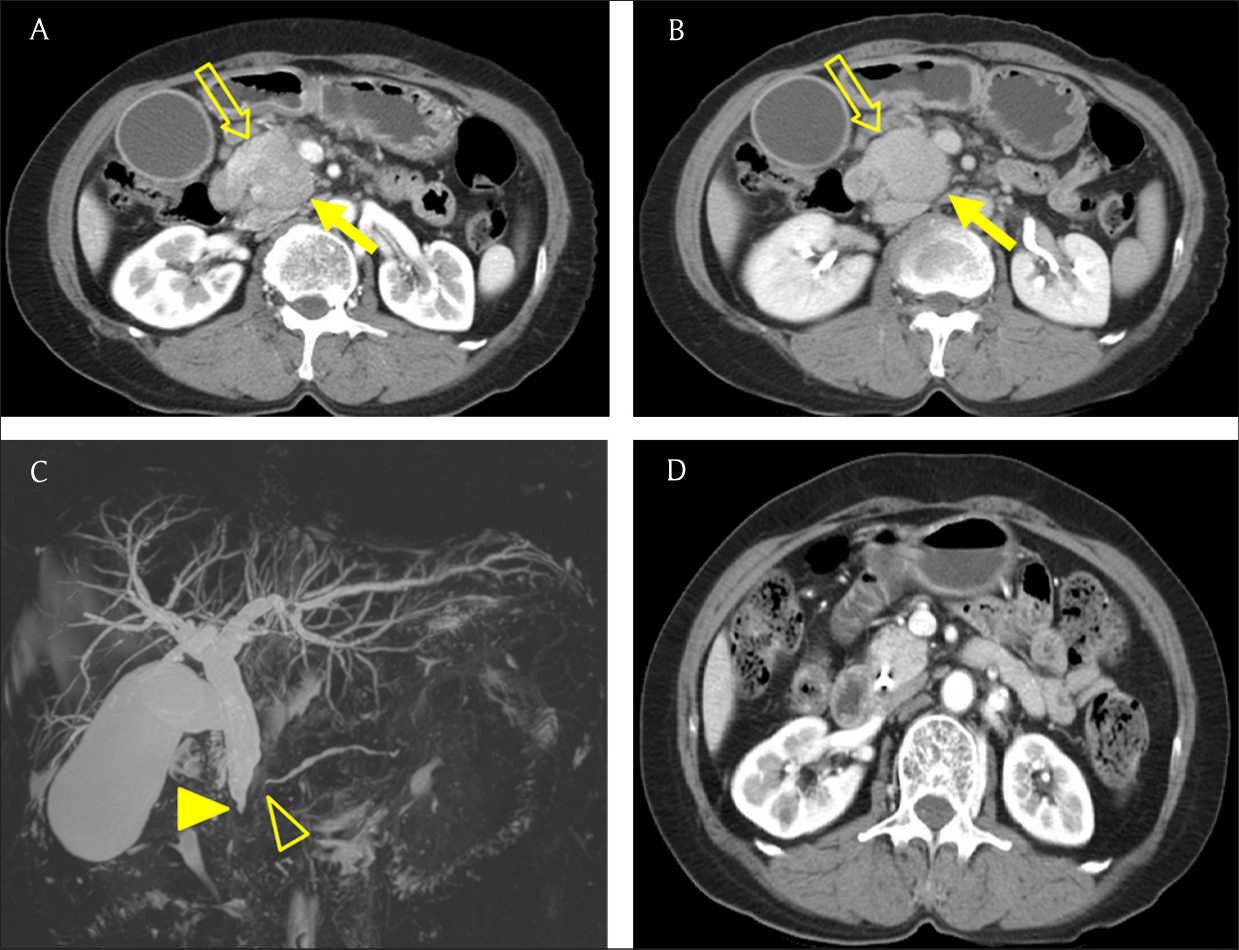
A 67-year-old woman with focal type autoimmune pancreatitis. **(A)** Axial pancreatic phase CT image shows a low attenuating mass (arrow) in the pancreatic head. Note the relatively high attenuation of the normal pancreatic parenchyma (open arrow). **(B)** On axial portal venous phase CT image, the mass (arrow) shows delayed enhancement and similar attenuation to the normal pancreas parenchyma (open arrow). **(C)** MRCP image shows dilatation of the bile and pancreatic ducts with smooth tapered narrowing (arrowhead and open arrowhead). **(D)** On axial portal venous phase CT image obtained after 50 days, the pancreatic lesion has resolved.

**Figure 7:**
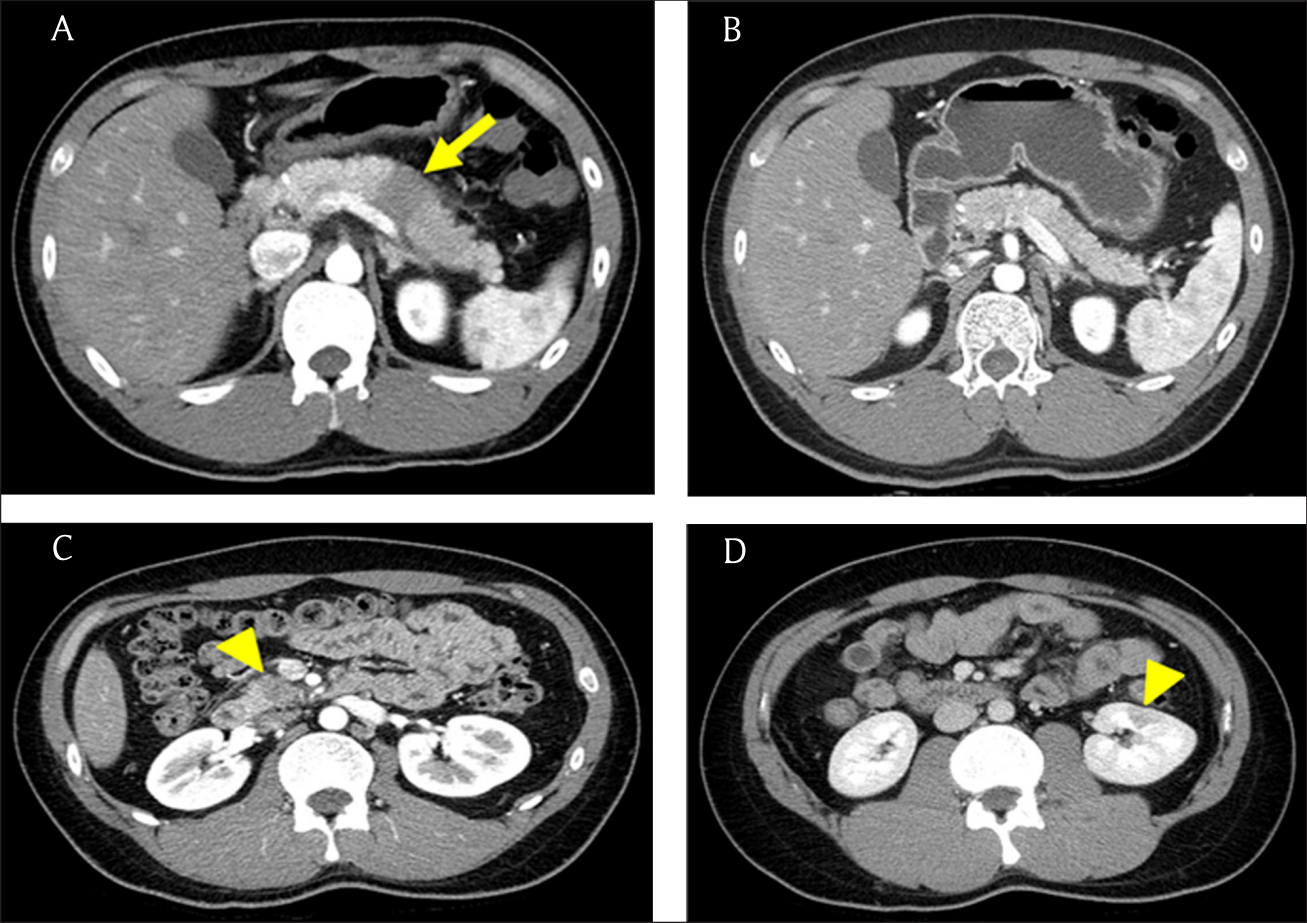
A 23-year-old man with focal type autoimmune pancreatitis. **(A)** Axial pancreatic phase CT image shows a low attenuating lesion (arrow) in the pancreatic body. Note no pancreatic duct dilatation in the tail portion. **(B)** Axial pancreatic phase CT image obtained after 3 months shows the disappearance of the mass. **(C, D)** On follow-up CT image obtained after seven months, newly developed low attenuating lesions (arrowheads) are noted in the uncinate process of the pancreas and the left kidney.

Autoimmune pancreatitis (AIP) is an uncommon form of chronic pancreatitis caused by an autoimmune mechanism [27]. It is a challenge to distinguish focal AIP from PDAC because the two diseases show similar imaging features, but several reports [28,29,30,31] have offered suggestions for discriminating between them. According to those studies, slightly lower or similar signal intensity compared with the spleen on unenhanced T1-weighted images, relatively homogeneous enhancement, signs of pancreatic duct penetration, smooth tapered narrowing of the pancreatic duct (icicle sign) or bile duct, multifocal stricture of the pancreatic duct, and a delayed enhancement pattern on dynamic enhanced images are features favoring AIP over PDAC (Figure 6). Another clue for diagnosis of AIP is involvement of an extra-pancreatic organ such as the biliary tree, retroperitoneum, salivary gland, or kidney (Figure 7) [8].

### Groove pancreatitis

**Figure 8:**
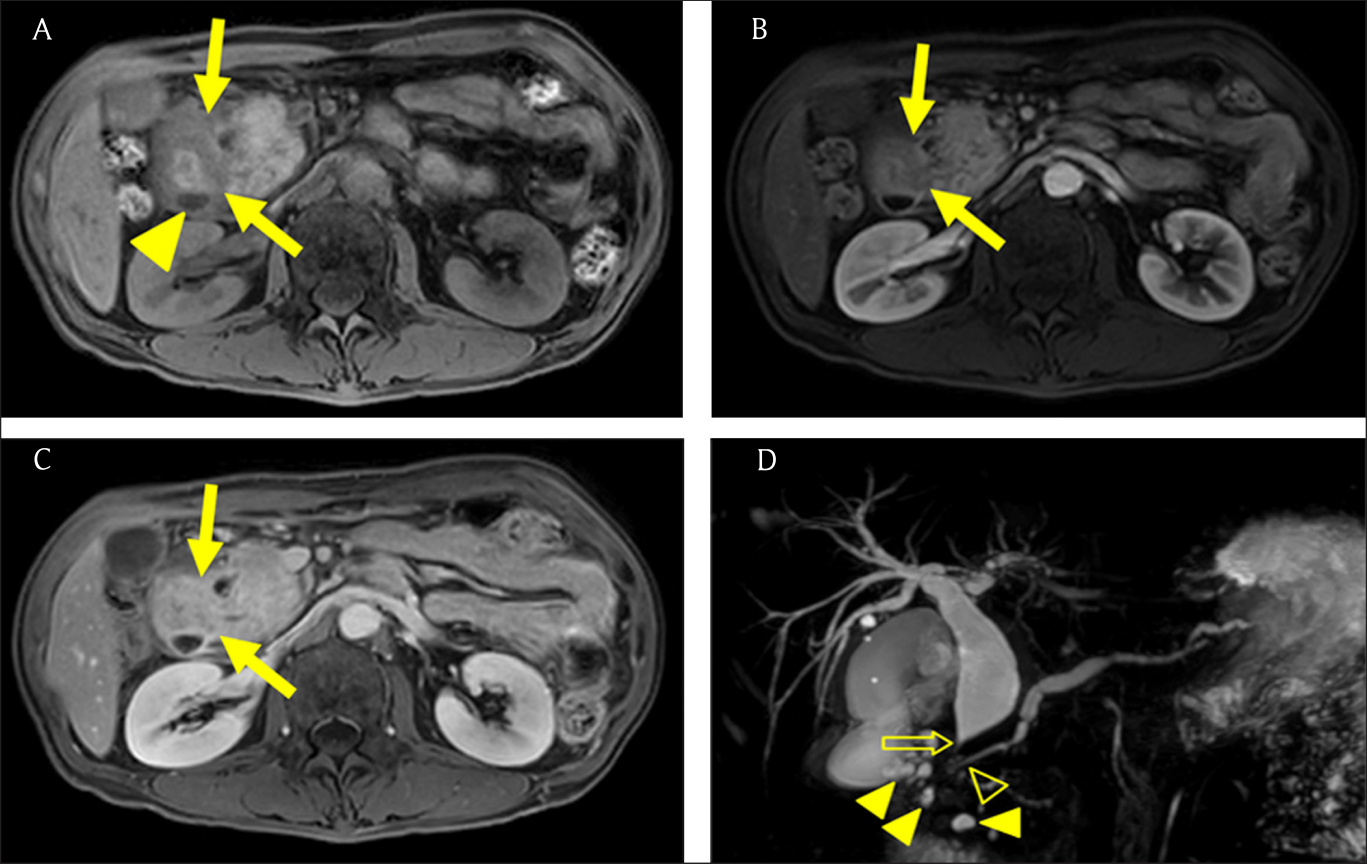
A 49-year-old man with groove pancreatitis who had a history of alcohol consumption greater than 50 g/day for more than 20 years. **(A)** Axial T1-weighted image shows a hypointense lesion (arrows) in the pancreaticoduodenal groove. There is an oval low signal intensity lesion (arrowhead) in the duodenal wall, suggesting a cyst. **(B, C)** Axial gadolinium-enhanced arterial and 3-minute delayed phase MR images show delayed enhancement of the mass-like lesion (arrows). **(D)** MRCP image shows dilatation of the bile and pancreatic ducts with smooth tapered narrowing (open arrow and open arrowhead). Note multiple cystic lesions (arrowheads) around the pancreaticoduodenal groove.

Groove pancreatitis is an uncommon type of pancreatitis affecting the pancreaticoduodenal groove, defined as the potential space between the pancreatic head, common bile duct, and duodenum [32]. This inflammatory lesion commonly develops in middle-aged men with a history of chronic alcoholism [32]. On imaging, groove pancreatitis presents as an ill-defined lesion between the pancreatic head and the duodenum, sometimes with bile and pancreatic duct narrowing; therefore, it can mimic PDAC (Figure 8) [9,25,33]. However, groove pancreatitis reveals a sheet-like curvilinear appearance and delayed enhancement. In addition, it is accompanied by cystic dystrophy in the duodenal wall and smooth bile duct narrowing (Figure 8D) [9,33].

## Conclusion

Neoplastic lesions such as high-grade NETs, small SPTs, and metastases and inflammatory lesions including focal AIP and groove pancreatitis can mimic PDAC. Abrupt narrowing of a dilated pancreatic duct is a usual imaging finding of PDAC. Although some mimics occasionally accompany pancreatic duct dilatation, they have points of differential diagnosis: presence of tumor thrombus and hypervascular liver metastases, absence of adjacent vascular invasion, and delayed enhancement pattern. In addition to these imaging findings, the shape of the narrowed pancreatic duct is a key imaging feature for discrimination of PDAC from other disease entities. Familiarity with the imaging features of PDAC and its mimics is paramount for managing patients in daily practice.

## Figures and Tables

**Table:** 

AIP	Autoimmune pancreatitis
CT	Computed tomography
MR	Magnetic resonance
MRCP	Magnetic resonance cholangiopancreatography
NET	Neuroendocrine tumor
PDAC	Pancreas ductal adenocarcinoma
SPT	Solid pseudopapillary tumor
